# Childhood health and educational disadvantage are associated with adult multimorbidity in the global south: findings from a cross-sectional analysis of nationally representative surveys in India and Brazil

**DOI:** 10.1136/jech-2022-219507

**Published:** 2023-08-04

**Authors:** Sanghamitra Pati, Abhinav Sinha, Priyanka Verma, Jayasingh Kshatri, Srikanta Kanungo, Krushna Chandra Sahoo, Pranab Mahapatra, Sandipana Pati, Felipe Mendes Delpino, Andria Krolow, Doralice Severo da Cruz Teixeira, Sandro Batista, Bruno P Nunes, David Weller, Stewart W Mercer

**Affiliations:** 1 Division of Public Health, ICMR-Regional Medical Research Centre, Bhubaneswar, Odisha, India; 2 Department of Psychiatry, Kalinga Institute of Medical Sciences, Bhubaneswar, Odisha, India; 3 Lown Fellow, Harvard T H Chan School of Public Health, Boston, Massachusetts, USA; 4 Department of Health & Family Welfare, Odisha State Institute of Health and Family Welfare, Bhubaneswar, Odisha, India; 5 Department of Nursing, Federal University of Pelotas, Pelotas, Brazil; 6 School of Medicine, Federal University of Goias, Goiania, Brazil; 7 College of Medicine, The University of Edinburgh Usher Institute of Population Health Sciences and Informatics, Edinburgh, UK

**Keywords:** CHILD HEALTH, CHRONIC DI, multimorbidity, India, Brazil

## Abstract

**Introduction:**

Multimorbidity has emerged as a major healthcare challenge in low/middle-income countries (LMICs) such as India and Brazil. Life course epidemiology suggests that adverse events in early life contribute to an individual’s later health in adulthood. However, little is known about the influence of early life health and social factors on the development of multimorbidity in adulthood in LMICs. We aimed to explore the association of adult multimorbidity with childhood health and social disadvantages among two LMICs, India and Brazil.

**Methods:**

We conducted a secondary data analysis of older adults aged ≥50 years using nationally representative surveys from Longitudinal Ageing Study in India, 2017–2018 (n=51 481) and ‘Estudo Longitudinal da Saude e Bem-Estar dos Idosos Brasileirous’, 2015–2016 (n=8730). We estimated the prevalence of multimorbidity along with 95% CI as a measure of uncertainty for all weighted proportions. Log link in generalised linear model was used to assess the association between childhood health and disadvantages with multimorbidity, reported as adjusted prevalence ratio (APR).

**Results:**

The prevalence of multimorbidity was 25.53% and 55.24% in India and Brazil, respectively. Participants who perceived their childhood health as poor and missed school for a month or more due to illness had the highest level of multimorbidity across both countries. After adjusting for age and gender, a significant association between adult multimorbidity and poor self-rated childhood health (APR: (India: 1.38, 1.16 to 1.65) and (Brazil: 1.19, 1.09 to 1.30)); and missed school for a month due to illness (AOR: (India: 1.73, 1.49 to 2.01) and (Brazil: 1.16, 1.08 to 1.25)) was observed.

**Conclusion:**

Early life health, educational and economic disadvantages are associated with adult multimorbidity and appear to contribute to the later course of life. A life course approach to the prevention of multimorbidity in adulthood in LMICs may be useful in health programmes and policies.

**WHAT IS ALREADY KNOWN ON THIS TOPIC**

- Among low/middle-income countries (LMICs), we identified only one study from China which investigated the association between childhood adversity and trajectories of multimorbidity in mid-late life using data from China Health and Longitudinal Retirement Survey.

**WHAT THIS STUDY ADDS**

- This is the first study to investigate the association of childhood health and disadvantages with adult multimorbidity in LMICs within nationally representative surveys.
- We observed a twofold prevalence of multimorbidity in Brazil than India. Our findings indicate self-reported inferior childhood health and participants who missed school for a month or more due to illness were strongly associated with adult multimorbidity uniformly across both nations.

**HOW THIS STUDY MIGHT AFFECT RESEARCH, PRACTICE OR POLICY**

- Our findings are consistent with the previous literature available from high-income countries (HICs) where early life health has been shown to be linked to an increased risk of multimorbidity in adulthood.
- This suggests a life course approach in combating multimorbidity is warranted in LMICs as well as HICs.
- Further, longitudinal studies or panel data analysis is required to support causality.

## Introduction

Multimorbidity, the coexistence of two or more chronic conditions within an individual (including long-term non-communicable diseases, mental health conditions and/or infectious diseases of long duration), has emerged as a significant healthcare challenge in low/middle-income countries (LMICs) requiring informed and comprehensive policy interventions.^1 2^ Owing to the demographic vis-à-vis epidemiological transition, multimorbidity has become a norm rather than exception in older age groups in LMICs such as India and Brazil. Recent evidence shows a high prevalence of multimorbidity among older adults aged ≥45 years to be around 34.5% in India and around 67.8% among Brazilians aged ≥50 years.^3 4^ Multimorbidity increases with age and contributes to health inequalities in younger age groups.^5^ It predisposes an individual to poor health, worsens health-related quality of life, increases healthcare utilisation and adversely affects physical functioning and mental health.^6–9^ Additionally, concurrent usage of multiple long-term drugs (polypharmacy) can reduce adherence and have other detrimental effects due to adverse drug reactions and interactions.^10^

Health is influenced by a series of correlated events such as lower educational attainment leading to low socioeconomic status referred as the ‘the long arm of childhood’; where childhood factors act as a catalyst for adult health.^11^ Evidence suggests that childhood adversities are linked to the development of chronic conditions such as hypertension and diabetes.^12 13^ Such evidence is based on the life course epidemiology (LCE) framework, which suggests that events in early life determine an individual’s later health.^14 15^ It can broadly be described by two theories, the first being the ‘critical period’ or ‘latency model’ in which early life conditions establish a biological imprint making an individual susceptible to compromised health states in adult life. Supporting this theory, a previous study established the linkage between childhood health and various chronic conditions in adulthood.^16^ The second explanation to LCE is through the ‘accumulation of risk model’, which states that early life events lead an individual to varying health pathways, through a series of connected events known as ‘chains of risk’. In this model, childhood conditions such as childhood socioeconomic status, or lesser years of schooling lead to lower income during later life, which may further cause health problems in adulthood.^17^

Given the increasing burden of multimorbidity in LMICs, it is imperative to understand the early life course origins of multimorbidity to potentially address modifiable risk factors. Previous studies in high-income countries (HICs) have shown that adult multimorbidity is associated with childhood adversities.^18–24^ However, to the best of our knowledge, this has not been explored in LMICs. It is important to explore evidence on linkages between childhood health conditions and disadvantages with adult multimorbidity in LMICs to formulate and strengthen the policies with a life course approach. The aim of the present study was to explore any association of multimorbidity with childhood health conditions and economic/educational disadvantage among two global southern LMICs, India and Brazil, using recent nationally representative data in both the countries.

## Methods

### Overview of data

This study used baseline data from Longitudinal Ageing Study in India (LASI), wave 1 and Estudo Longitudinal da Saude e Bem-Estar dos Idosos Brasileirous (ELSI-Brazil). Both LASI and ELSI are longitudinal ageing studies based on a nationally representative survey. LASI wave 1 was conducted in the year 2017–2018 and it included respondents aged 45 years and above along with their spouses (irrespective of age), whereas ELSI was conducted in the year 2015–2016 and included respondents aged 50 years and above. LASI wave 1 interviewed a total of 72 250 individuals from 29 states and six union territories of India, while ELSI interviewed 9412 individuals inhabiting 70 municipalities across five different geographic areas in Brazil. The detailed methodologies for LASI and ELSI are published on their respective websites.^25 26^

### Study population and sample size

**Figure 1:**
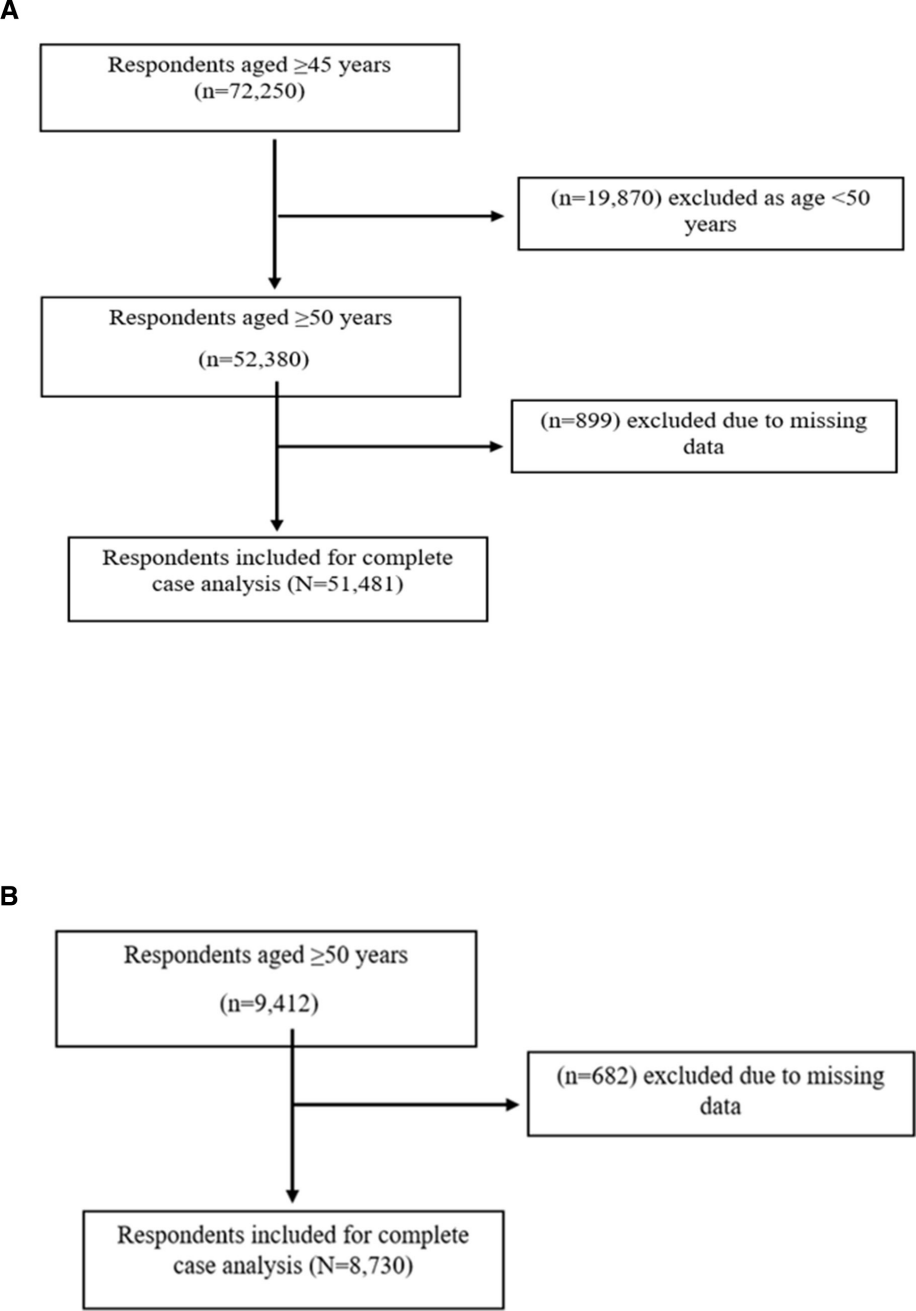
Sample flowchart for (A) LASI and (B) ELSI. ELSI, Estudo Longitudinal da Saude e Bem-Estar dos Idosos; LASI, Longitudinal Ageing Study in India.

We excluded participants aged less than 50 years from the LASI cohort (figure 1A), since ELSI only included participants aged 50 years and above. We also excluded 899 participants from LASI and 682 respondents from ELSI whose data were missing on some or the other variable. The study thus included 51 481 respondents from LASI and 8730 respondents from ELSI to perform a complete case analysis. A flowchart summarising the sample of respondents corresponding to both countries is shown in figure 1A and B.

### Variable selection

#### Exposure variables

Self-rated childhood health, educational disadvantages and childhood economic status of the family were considered as exposure variables. Participants were asked to rate their childhood health as very good, good, fair and poor. No formal education and missed school for a month or more due to health issues was considered as an educational disadvantage. Family’s economic status during childhood was self-reported as pretty well off, average, poor and financial status varied a lot. The detailed description of each indicator presented in LASI and ELSI is provided as online supplemental tables S1 and S2, respectively.

10.1136/jech-2022-219507.supp1 Supplementary data

#### Outcome variable

Multimorbidity, our main outcome of interest, was defined as the count of two or more chronic conditions within a single individual. We included 11 self-reported chronic conditions which were uniform in both LASI and ELSI for our analysis classified as follows hypertension; diabetes; stroke; cancer; chronic lung diseases; chronic heart diseases; bone/joint diseases; neurological or psychiatric problems; high cholesterol; chronic renal failure and chronic oral conditions. These conditions were chosen based on an extensive review of the existing literature.^1 4 9^ More details on all chronic conditions from LASI and ELSI can be found in online supplemental tables S1 and S2, respectively. In accordance with our objectives, data on chronic conditions were categorised into two conditions: multimorbidity present and multimorbidity absent.

### Covariates

We included the following covariates for analysis: age (50–59, 60–69, >70 years), gender (male/female), residence (urban/rural), education (no formal education, less than primary, primary and middle completed, secondary and higher up to diploma, graduation and above), occupation (currently working/currently not working), marital status (have partner/does not have partner) and wealth index (poorest, poorer, middle, richer, richest). For LASI, the wealth index was based on monthly per capita expenditure while for ELSI we performed principal component analysis based on the household assets to form wealth quintiles. The detailed description of each variable used from LASI and ELSI is provided in online supplemental tables S1 and S2, respectively.

### Statistical analysis

Data were analysed using STATA V.17.0 (StataCorp, Texas, USA) statistical software package. Prevalence of multimorbidity was estimated for both countries and descriptive statistics was used to evaluate the sample characteristics, expressed in terms of frequency and proportion (n, %). Additionally, a 95% CI was reported as a measure of uncertainty for all weighted proportions. We used a log link in generalised linear binomial model to estimate the associations since the prevalence of multimorbidity was high which may deviate ORs from their actual relative risk. To examine the association of multimorbidity with childhood health and disadvantage with various demographic characteristics, a pairwise bivariate association was investigated, expressed as prevalence ratio (PR) as a strength of association. We then adjusted the model for age and sex. The risk measure for this was reported as an adjusted prevalence ratio (APR) with 95% CI to see the association between variables. All analysis was done considering sample weights to compensate for the complex survey design.

### Ethical consideration

The present study is a type of secondary data analysis and does not involve any risk to the human participant. We used anonymous secondary data from LASI and ELSI hence, ethical permission was not required. Both the original studies received ethical clearance from their respective ethical review committees and incorporated informed consent from the participants prior to the interview.

### Patient and public involvement

Patient or the public were not involved in the design, or conduct, or reporting, or dissemination plans of our research.

## Results

While the distribution of most of the sociodemographic characteristics was similar in both datasets, the majority of respondents were urban residents in the ELSI compared with the majority of rural in LASI as shown in table 1.

**Table 1:** Weighted characteristics of the study population

Correlates	India (N=51 481), n (%)	Brazil (N=8730), n (%)
Age (in years)		
50–59	19 835 (38.53)	4245 (48.63)
60–69	18 807 (36.53)	2603 (29.82)
≥70	12 839 (24.94)	1882 (21.55)
Sex		
Male	23 942 (46.51)	4031 (46.17)
Female	27 539 (53.49)	4699 (53.83)
Residence		
Urban	15 835 (30.76)	7415 (84.93)
Rural	35 646 (69.24)	1315 (15.07)
Education		
No education	27 289 (53.01)	1132 (12.97)
Less than primary	5696 (11.06)	3301 (37.81)
Primary and middle completed	10 060 (19.54)	2011 (23.04)
Secondary and higher up to diploma	6032 (11.72)	1695 (19.41)
Graduation and above	2404 (4.67)	591 (6.77)
Occupation		
Currently working	21 870 (42.48)	2775 (31.79)
Currently not working	29 611 (57.52)	5955 (68.21)
Marital status		
Have partner	36 502 (70.90)	5602 (64.17)
No partner	14 978 (29.10)	3128 (35.83)
Wealth Index		
Poorest class	10 974 (21.32)	1857 (21.27)
Poorer class	11 017 (21.40)	1844 (21.13)
Middle class	10 491 (20.37)	1752 (20.07)
Richer class	9920 (19.27)	1657 (18.98)
Richest class	9079 (17.64)	1620 (18.55)
*Childhood health conditions and disadvantages*		
Self-rated childhood health		
Very good	25 044 (48.65)	2155 (24.68)
Good	19 979 (38.81)	4683 (53.64)
Fair	5640 (10.95)	1327 (15.20)
Poor	818 (1.59)	565 (6.48)
Missed school for a month or more due to health issues		
Never attended school	27 288 (53.01)	1750 (20.00)
Missed school	1014 (1.97)	746 (8.55)
Not missed school	23 179 (45.02)	6234 (71.41)
Childhood economic status		
Pretty well off	4054 (7.87)	455 (5.21)
Average	25 309 (49.16)	1958 (22.43)
Poor	21 717 (42.19)	4997 (57.24)
Varied	401 (0.78)	1320 (15.12)

**Figure 2:**
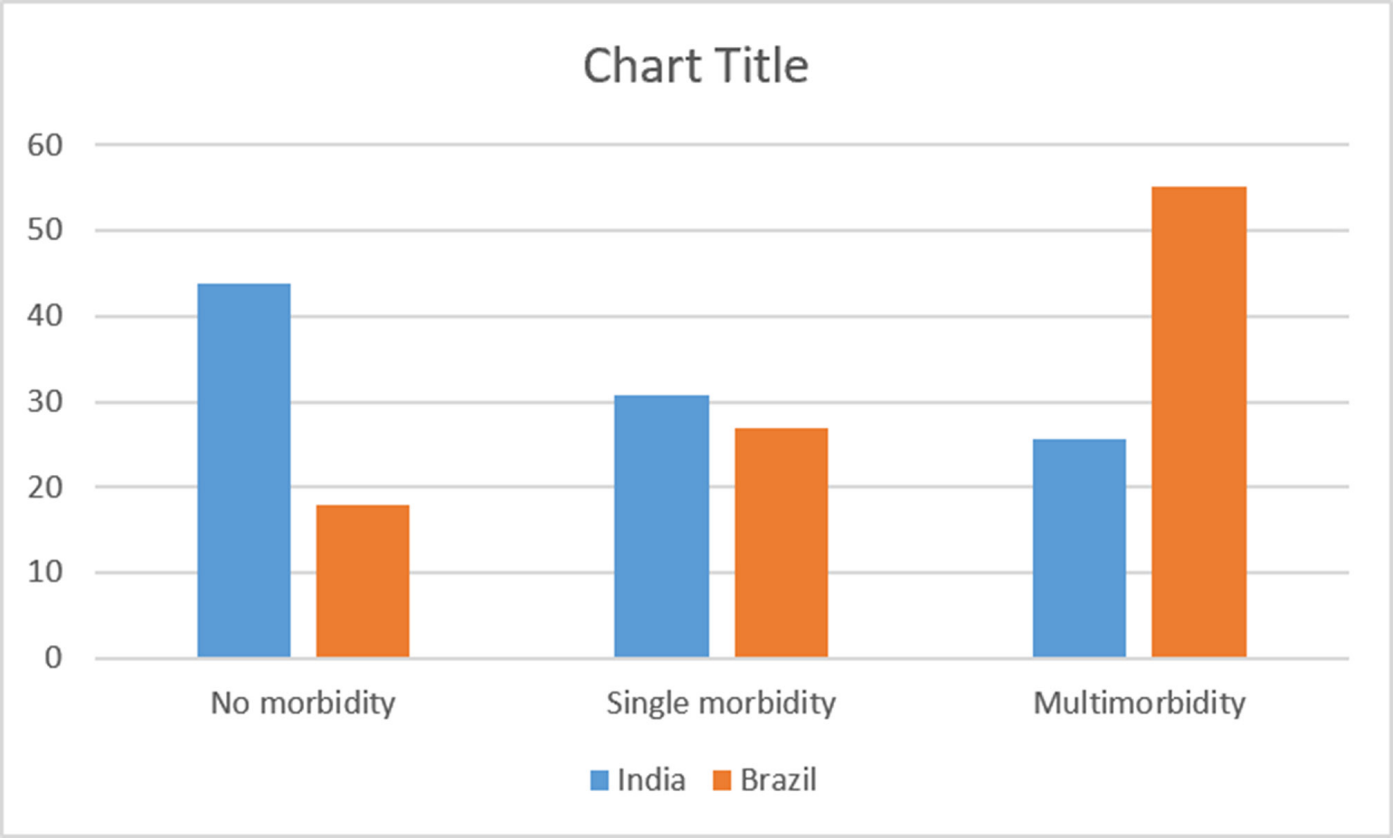
Morbidity status across study population.

Very few respondents rated their childhood health as poor in LASI (1.59%) and ELSI (6.48%). The proportion of respondents who did not receive any education was found to be around 53% and 13% across LASI and ELSI, respectively. 42.19% of the LASI population and 57.24% of the ELSI population reported their childhood economic status as poor. Hypertension was the leading chronic condition across both the countries. The distribution of various chronic conditions is presented in online supplemental table S3. The prevalence of multimorbidity was found to be 25.53% in India and 55.24% in Brazil (figure 2).

In both countries, multimorbidity was more prevalent among females, urban residents, those currently not working and who lived without partners (table 2). The prevalence of multimorbidity among those who received no formal education was lower (21.7%) compared with the same group in Brazil (55.6%). Higher prevalence of multimorbidity was found among respondents who rated their childhood health as poor in comparison to their counterparts, across both the countries. Among those who rated their childhood economic status as poor, the prevalence of multimorbidity was 21.47% in India and 57.52% in Brazil (table 2).

**Table 2:** Prevalence and distribution of multimorbidity across sociodemographic characteristics

Demographic characteristics		India (LASI)		Brazil (ELSI)	
		Weighted prevalence of multimorbidity % (95% CI)	Prevalence ratio (95% CI)	Weighted prevalence of multimorbidity % (95% CI)	Prevalence ratio (95% CI)
Age	50–59 years	21.19 (20.62 to 21.76)	Ref	47.79 (46.28 to 49.31)	Ref
	60–69 years	27.39 (26.75 to 28.03)	1.29 (1.17 to 1.43)	61.28 (59.37 to 63.15)	1.28 (1.21 to 1.35)
	≥70 years	29.51 (28.72 to 30.31)	1.39 (125 to 1.55)	63.66 (61.46 to 65.87)	1.33 (1.26 to 1.41)
Sex	Male	23.68 (23.14 to 24.23)	Ref	44.80 (43.27 to 46.36)	Ref
	Female	27.13 (26.61 to 27.66)	1.14 (1.06 to 1.24)	64.19 (62.80 to 65.56)	1.43 (1.36 to 1.50)
Residence	Rural	21.45 (21.03 to 21.88)	Ref	49.41 (46.70 to 52.17)	Ref
	Urban	34.70 (33.96 to 35.45)	1.62 (1.49 to 1.76)	56.27 (55.13 to 57.40)	1.14 (1.06 to 1.22)
Education	Graduation and above	28.30 (26.49 to 30.13)	Ref	52.17 (48.00 to 56.21)	Ref
	High school/intermediate	34.70 (33.50 to 35.91)	1.23 (0.99 to 1.51)	51.31 (48.92 to 53.73)	0.98 (0.88 to 1.10)
	Middle school complete	28.67 (27.78 to 29.56)	1.01 (0.84 to 1.21)	55.05 (52.84 to 57.24)	1.05 (0.94 to 1.18)
	Up to primary school	27.39 (26.23 to 28.56)	0.97 (0.81 to 1.15)	57.77 (56.06 to 59.46)	1.11 (1.00 to 1.23)
	No formal education	21.71 (21.22 to 22.21)	0.77 (0.65 to 0.91)	55.64 (52.70 to 58.57)	1.07 (0.95 to 1.19)
Occupation	Currently working	17.00 (16.50 to 17.50)	Ref	45.27 (43.40 to 47.13)	Ref
	Currently not working	31.83 (31.30 to 32.36)	1.87 (1.71 to 2.04)	59.88 (58.62 to 61.13)	1.32 (1.25 to 1.40)
Marital status	Have partners	24.80 (24.36 to 25.25)	Ref	53.56 (52.24 to 54.87)	Ref
	Does not have partners	27.29 (26.58 to 28.01)	1.10 (1.01 to 1.20)	58.24 (56.50 to 59.98)	1.09 (1.04 to 1.14)
Wealth index	Poorest class	18.75 (18.02 to 19.49)	Ref	57.94 (55.66 to 60.20)	Ref
	Poorer class	21.90 (21.12 to 22.68)	1.17 (1.07 to 1.28)	56.23 (54.05 to 58.62)	0.97 (0.90 to 1.04)
	Middle class	24.65 (23.83 to 25.49)	1.31 (1.19 to 1.45)	58.89 (56.55 to 61.30)	1.02 (0.95 to 1.09)
	Richer class	28.16 (27.28 to 29.06)	1.50 (1.35 to 1.67)	50.72 (48.32 to 53.19)	0.87 (0.81 to 0.94)
	Richest class	36.27 (35.28 to 37.27)	1.93 (1.72 to 2.18)	51.56 (49.08 to 54.00)	0.89 (0.83 to 0.96)
Self-rated childhood health	Very good	24.87 (24.33 to 25.41)	Ref	52.52 (50.42 to 54.68)	Ref
	Good	25.10 (24.50 to 25.70)	1.01 (0.93 to 1.10)	52.75 (51.30 to 54.18)	1.00 (0.95 to 1.07)
	Fair	28.64 (27.46 to 29.84)	1.15 (0.98 to 1.35)	62.10 (59.42 to 64.71)	1.18 (1.10 to 1.27)
	Poor	34.66 (31.34 to 37.97)	1.39 (1.19 to 1.63)	70.01 (66.13 to 73.83)	1.33 (1.23 to 1.44)
Missed school for a month or more due to health issues	Never attended school		Ref	56.65 (54.27 to 58.97)	Ref
	Missed school	35.25 (32.26 to 38.23)	1.62 (1.42 to 1.86)	64.30 (60.69 to 67.70)	1.13 (1.05 to 1.23)
	Not missed school	29.60 (29.01 to 30.19)	1.36 (1.26 to 1.47)	53.75 (52.51 to 55. 00)	0.95 (0.90 to 1.00)
Childhood economic status	Pretty well off	32.97 (31.53 to 34.45)	1.53 (1.31 to 1.79)	54.80 (50.02 to 59.36)	0.95 (0.86 to 1.06)
	Average	27.81 (27.25 to 28.36)	1.29 (1.20 to 1.39)	53.22 (50.98 to 55.45)	0.92 (0.87 to 0.98)
	Poor	21.47 (20.92 to 22.02)	Ref	57.52 (56.13 to 58.89)	Ref
	Varied	26.27 (21.95 to 30.78)	1.22 (0.91 to 1.64)	49.74 (47.04 to 52.50)	0.86 (0.80 to 0.93)

ELSI, Estudo Longitudinal da Saude e Bem-Estar dos Idosos; LASI, Longitudinal Ageing Study in India.

As shown in table 2, in both countries, we observed multimorbidity to be associated with greater PRs among higher age groups, and female gender. Individuals with poor childhood health were at a greater risk of having multimorbidity in adulthood in both India (PR: 1.39; 95% CI:1.19 to 1.63) and Brazil (PR: 1.33; 95% CI: 1.23 to 1.44). Similarly, participants who missed school for a month or more had a higher risk of having multimorbidity (PR: 1.62; 95% CI: 1.42 to 1.86) and (PR: 1.13; 95% CI: 1.05 to 1.23) for India and Brazil, respectively.

In multivariate analyses, after adjusting for age and gender, we found that poor self-rated childhood health and missing school for a month or more due to health issues were significantly associated with a higher risk of adult multimorbidity in both India and Brazil (table 3). However, individuals with well-off childhood economic status were significantly more likely to have multimorbidity in adulthood (APR: 1.32; 95% CI: 1.14 to 1.53) in India.

**Table 3:** Multivariate analysis between multimorbidity and self-reported childhood health and disadvantages (adjusted for age and gender)

Demographic characteristics	Multimorbidity			
	India		Brazil	
	APR	95% CI	APR	95% CI
Self-rated childhood health				
Very good	Ref		Ref	
Good	1.03	0.95 to 1.12	0.99	0.94 to 1.05
Fair	1.17	1.00 to 1.38	1.15	1.07 to 1.23
Poor	1.38	1.16 to 1.65	1.19	1.09 to 1.30
Never attended school/missed school for a month or more due to health issues				
Never attended school	Ref		Ref	
Missed school	1.73	1.49 to 2.01	1.16	1.08 to 1.25
Not missed school	1.50	1.36 to 1.65	1.02	0.97 to 1.08
Childhood economic status				
Poor	Ref		Ref	
Average	1.21	1.13 to 1.30	0.97	0.92 to 1.02
Pretty well off	1.32	1.14 to 1.53	0.93	0.84 to 1.03
Varied	1.24	0.95 to 1.62	0.93	0.87 to 0.99

APR, adjusted prevalence ratio.

## Discussion

This study used data from respondents, aged 50 years or more, from nationally representative surveys in India and Brazil to explore the association between childhood health, educational and economic disadvantages, and adult multimorbidity status. We found that the prevalence of multimorbidity was over twice as high in Brazil compared with India. Our analyses suggest that multimorbidity is significantly associated with increasing age, higher wealth quintiles and female gender. Both bivariate and multivariate analyses adjusted for age and gender show that self-rated poor childhood health and missing school (for at least 1 month) due to health-related issues was associated with increased odds of adult multimorbidity. Individuals with well-off childhood economic status were significantly more likely to have multimorbidity in adulthood in India, but not in Brazil.

Other community-based studies among those over 60 years of age have reported the prevalence of multimorbidity to be around 81% in Brazil and 49% in India compared with 62% and 28% in our study in the same age group.^27 28^ This difference can probably be attributed to the specific inclusion criteria (rural population/primary care settings, for example) and the methodology used in these surveys (self-reported vs use of screening tools). The association of age with multimorbidity in our study is consistent with the international literature.^29^ The difference in the prevalence of multimorbidity between India and Brazil is probably due to the fact that ELSI included predominantly urban respondents as compared with LASI, who might have a better access to healthcare, screening and diagnostic services, and more health literacy. Also, Gross Domestic Product (GDP) per capita in Brazil is four times greater than India which makes it more difficult to compare the two countries directly.^30^ This is also reflected by sample characteristics where we observed India had 53% of participants with no formal education compared with only 13% in Brazil.

Ours is the first study to report a significant association between poor childhood health and adult multimorbidity in LMICs though others have established similar findings in HICs.^18–24^ It is challenging to compare our findings with studies in similar settings as most literature on the topic is from HIC. Also, a lower percentage of individuals in the study, reporting poor childhood health may be an indicative of differential survival, where those who had poor health during childhood may have been less likely to survive to adulthood. However, it is important to recognise that health in the earliest years of life has an enormous impact on the development of biological systems that enables children to thrive and grow up to be a healthy adult.^31^ Childhood experiences form the social, emotional and physical skills needed for a healthy lifestyle throughout the life course, thereby influencing chronic lifestyle disorders in adulthood.^32 33^ Similarly, childhood socioeconomic status is known to influence the exposure to stress,^34^ thereby impacting adult health.

However, we found individuals with affluent childhood economic status were more likely to have multimorbidity in India, which was in contrast to the findings of Brazil. The findings from India are consistent with previous Indian studies which report multimorbidity to be more akin among the affluent class. An explanation to this could be the change in lifestyle including dietary habits, reduced physical activity etc., with an increased income among the affluent Indians. However, previous studies in HICs contradict our findings, where childhood financial hardships were found to be positively associated with multimorbidity.^35^ Moreover, India and Brazil are at different stages of the demographic and epidemiological transition which could be one of the other reasons for this difference. Additionally, nutritional differences may also explain this phenomenon as previous India studies suggest that higher body mass index is associated with higher socioeconomic status.^36^ This stands true for many LMICs such as India where people start eating unhealthy food as they have money which gradually leads to increased risk of multimorbidity. However, this requires further exploration.

### Implications for policy and practice

Our findings suggest childhood health affects later life, which warrants a life course approach in the formulation and implementation of programmes and policies. The holistic approach (targeting the social determinants of health such as education, poverty and childhood health) will help in approaching towards a life course approach instead of isolated approaches will help in adopting preventive measures in early life. This will also enhance the quality of life in the later stages.

### Strengths and limitations

We used nationally representative data of two global southern LMICs with big sample size which is a major strength of this study. This is the first ever study to report the association of childhood health conditions and disadvantages with multimorbidity. Our study is limited by the use of self-rated responses for childhood health up to 16 years of age, which is susceptible to recall bias. Furthermore, chronic conditions for multimorbidity assessment were self-reported. Additionally, our datasets are cross-sectional, hence causality could not be established. The use of chronic conditions with varying characteristics/different nature in the construction of the outcome variable, multimorbidity, may affect the findings of the study.

## Conclusion

Early life health and educational disadvantages are associated with adult multimorbidity and appear to contribute to the later course of life. Moreover, we observed economic position also contributed to multimorbidity in India. A life course approach to the prevention of multimorbidity in adulthood in LMICs may be useful in health programmes and policies.

## Data Availability

Data are available upon reasonable request.
