# Correction: *Childhood health and educational disadvantage are associated with adult multimorbidity in the global south: findings from a cross-sectional analysis of nationally representative surveys in India and Brazil*

**DOI:** 10.1136/jech-2022-219507corr1

**Published:** 2024-03-08

Pati S, Sinha A, Verma P, *et al*. Childhood health and educational disadvantage are associated with adult multimorbidity in the global south: findings from a cross-sectional analysis of nationally representative surveys in India and Brazil. *J Epidemiol Community Health* 2023;77:617–24.

Below are corrected and additional information related to the original version of this paper:

- ELSI-Brazil full name: ‘The Brazilian Longitudinal Study of Aging (ELSI-Brazil).’ The study’s full name had a few errors throughout the text.
- ‘Doralice Severo da Cruz Teixeira’ affiliation: School of Public Health, University of São Paulo, São Paulo, Brazil.
- ‘Sandro Batista’ affiliations:
  1. Department of Internal Medicine, School of Medicine, Federal University of Goiás, Goiânia, Brazil.
  2. Federal District Health Department, Brasília, Brazil.
- Additional ELSI-Brazil information: ELSI-Brazil was supported by the Brazilian Ministry of Health: DECIT/SCTIE (Grants: 404965/2012–1 and TED 28/2017); COPID/DECIV/SAPS (Grants: 20836, 22566, 23700, 25560, 25552, and 27510). ELSI-Brazil was approved by the Fundação Oswaldo Cruz (FIOCRUZ) ethics committee, Minas Gerais, Brazil (protocol number 34649814.3.0000.5091).

