# Supplementary material for: Childhood health and educational disadvantage are associated with adult multimorbidity in the global south: findings from a cross-sectional analysis of nationally representative surveys in India and Brazil
Source: J Epidemiol Community Health. 2023 Aug 4;77(10):617–24. doi: 10.1136/jech-2022-219507 (PMC10511991; doi:10.1136/jech-2022-219507)
Supplement: Supplementary data [file jech-2022-219507supp001.pdf]

Supplementary Files

Supplementary Table S1. Description of the various indicators included in the study, Longitudinal Ageing Study in India (LASI).

Indicators	Question	Options	Method (Categories)
Age	How old were you at your last birthday? (Age in complete years)	Continuous In years	50-59 years<br>60-69 years<br>≥70 years
Sex	Record sex of the respondent. If not clear, please ask the respondent.	1.Male<br>2.Female	Male<br>Female
Residence	Where have you lived most of your adult life?	1.Rural<br>2.Urban	Rural<br>Urban
Education	Have you ever attended school?	1.Yes<br>2. No (recoded as no formal education)	Recoded into ordered categories:<br>• No formal education<br>• Less than Primary (option 1)<br>• Primary and middle Completed (merged option 2-3)<br>• Secondary and higher up to Diploma (merged option 4-6)<br>• Graduation and above (merged option 7-9)
	If yes, what is the highest level of education that you have completed?	1.Less than Primary school (Standard 1-4)<br>2. Primary school Completed (Standard 5-7)<br>3. Middle school Completed (Standard 8- 9)<br>4. Secondary School/Matriculation completed<br>5. Higher Secondary/Intermediate/Senior Secondary completed<br>6. Diploma and certificate holders<br>7. Graduate degree (B.A., B.Sc., B. Com.) completed<br>8. Post-graduate degree or (M.A., M.Sc., M. Com.) above (M.Phil, Ph.D.,Post-Doc) completed<br>9. Professional course/degree (B.Ed, BE, B.Tech, MBBS, BHMS, BAMS, B. Pharm, BCS, BCA, BBA, LLB, BVSc., B. Arch, M.Ed, ME, M.Tech, MD, M.Pharm, MCS, MCA, MBA, LLM, MVSc., M. Arch, MS, CA, CS, CWA)	
Occupation	Are you currently working?	1.Yes<br>2.No	No: Currently not working

			Yes: Currently working
Marital status	What is your current (latest) marital status?	1.Currently married<br>2. Widowed<br>3. Divorced<br>4. Separated<br>5. Deserted<br>6. Live-in relationship<br>7. Never married	Recoded into:<br>• Have partner (merged option 1 and 6)<br>• No partner (merged option 2, 3, 4, 5 and 7)
Wealth index	Based on monthly per capita expenditure (MPCE)	Already categorized in dataset	• Poorest<br>• Poorer<br>• Middle<br>• Richer<br>• Richest
Self-rated childhood health	In general, would you say your childhood health was very good, good, fair, poor or very poor on the basis of what you remember, or what you heard or perceived from your parents?	1.Very good<br>2. Good<br>3. Fair<br>4. Poor<br>5. Very poor	Recoded as:<br>• Very good<br>• Good<br>• Fair<br>• Poor (merged poor and very poor)
Missed school for a month or more due to health issues	When you were growing up, before you were 16 years old, did you ever miss a month or more of school because of a health problem?	1. Yes<br>2. No	Recoded as:<br>• Missed school (option 1)<br>• Not missed school (option 2)
Childhood economic status	Now think about your family when you were growing up, from birth to age 16. Compared to other families in your community, would you say your family during that time was pretty well off financially, about average, or poor?	1. Pretty well off financially<br>2. Average<br>3. Poor<br>4. Varied	Recoded as:<br>• Poor (option 3)<br>• Average (option 2)<br>• Pretty well off (option 1)<br>• Varied (option 4)
Multimorbidity	Has any health professional ever diagnosed you with the following chronic conditions or diseases?<br>1. Hypertension or high blood pressure<br>2. Diabetes or high blood sugar<br>3. Cancer or a malignant tumor<br>4. Chronic lung disease (asthma ,chronic obstructive pulmonary disease/Chronic bronchitis or other chronic lung problems)<br>5. Chronic heart diseases (heart attack, congestive heart failure, or other chronic heart problems)	1. Yes<br>2. No	

	6. Stroke<br>7. Bone/joint diseases (Arthritis or rheumatism, Osteoporosis)<br>8. Any neurological, or psychiatric problems (depression , Alzheimer's, Parkinson's)<br>9. High cholesterol<br>10. Oral health (bleeding gums and swelling gums)		
	11. Have you ever been diagnosed with any of the following urogenital conditions or diseases? a. Have you ever been diagnosed with any of the following urogenital conditions or diseases?	a. Chronic renal failure	

Supplementary Table S2. Description of the various indicators included in the study, ELSI.

Indicators	Question	Options	Method (Categories)
Age	Age at the interview	Continuous In years	50-59 years<br>60-69 years<br>≥70 years
Sex	Sex	1.Male<br>2.Female	Male<br>Female
Residence	Residence	1.Rural<br>2.Urban	Rural<br>Urban
Education	What was the highest grade in school that you completed?	1. Never studied<br>2. 1st Grade (Elementary School)<br>3. 2nd Grade<br>4. 3rd Grade<br>5. 4th Grade<br>6. 5th Grade<br>7. 6th Grade (Middle School)<br>8. 7th Grade<br>9. 8th Grade<br>10. 9th Grade (High School)<br>11. 10th Grade<br>12. 11/12th Grade<br>13. GED<br>14. Some college (incomplete)	Recoded into ordered categories:<br>• No education (option 1)<br>• Less than Primary (merged option 2-5)<br>• Primary and middle Completed (merged option 6-9)<br>• Secondary and higher up to Diploma (merged option 10-14)<br>• Graduation and above (merged option15-18)

		15. College complete<br>16. Specialization/medical residency<br>17. Master's degree<br>18. Doctoral degree/PhD	
Occupation	Have you worked with a monetary compensation in the LAST 30 DAYS?	1. No<br>2. Yes	No: Currently not working<br>Yes: Currently working
Marital status	What is your current marital status?	1. Single<br>2. Married/ Common-law marriage/live together<br>3. Divorced or separated<br>4. Widow(er)	Recoded into:<br>• Have partner (option 2)<br>• No partner (merged option 1, 3 and 4)
Wealth index	Based on the household assets	Fridge<br>Washing machine<br>Dryer<br>Dish washer<br>Microwave<br>Television<br>VCR<br>Landline phone<br>Mobile phone<br>Air condition<br>Computer<br>Internet<br>Bike<br>Cable<br>Car	Through principal component analysis recoded as:<br>• Poorest<br>• Poorer<br>• Middle<br>• Richer<br>• Richest
Self-rated childhood health	Would you say that your health, since your BIRTH TO 15 YEARS OF AGE, was: (h11)	1. Excellent or very good<br>2. Good<br>3. Fair<br>4. Bad	Recoded as:<br>• Very good<br>• Good<br>• Fair<br>• Poor (Bad renamed as poor)
Missed school for a month or more due to health issues	Up to 15 YEARS OF AGE, were you absent from school FOR A MONTH OR LONGER due to health issues?	0. No<br>1. Yes<br>2. Did not go to school until 15 years old	Recoded as:<br>• Missed school (option 1)<br>• Not missed school (option 0)<br>• Never attended school (option 2)
Childhood economic status	Considering your childhood, since your BIRTH TO 15 YEARS OF AGE, you would say that your family: (h10)	1. Was pretty well off financially (above average)<br>2. Was about average<br>3. Was poor	Recoded as:<br>• Poor (option 3)<br>• Average (option 2)<br>• Pretty well off (option 1)<br>• Varied (option 4)

		4. The financial status varied a lot	
Multimorbidity	1. Hypertension<br>Has any doctor ever told you that you have hypertension (high blood pressure)?	0. No<br>1. Yes<br>2. Yes, only during pregnancy	Option 2 was excluded
	2. High cholesterol<br>Has any doctor ever told you that you have high cholesterol?	0. No<br>1. Yes	
	3. Stroke<br>Has a doctor ever told you that you had a cerebral vascular accident (stroke)?	0. No<br>1. Yes	
	4. Cancer<br>Has a doctor ever told you that you have or had cancer?	0. No<br>1. Yes	
	5. Chronic renal failure<br>Has a doctor ever told you that you have chronic renal failure?	0. No<br>1. Yes	
	6. Diabetes<br>Has any doctor ever told you that you have diabetes ("high blood sugar")?	0. No<br>1. Yes<br>2. Yes, only during pregnancy	Option 2 was excluded
	7. Chronic lung diseases by collapsing:<br>• Has a doctor ever told you that you have asthma? Exclude bronchitis<br>• Has a doctor ever told you that you have emphysema, chronic bronchitis, or chronic obstructive pulmonary disease (COPD)?	0. No<br>1. Yes	
	8. Neurological, or Psychiatric problems by collapsing:<br>• Has a doctor ever told you that you have depression?<br>• Has a doctor ever told you that you have Parkinson's disease?<br>• Has a doctor ever told you that you have Alzheimer's disease?	0. No<br>1. Yes	
	9. Bone/joint diseases by collapsing:<br>• Have a doctor ever told you that you have arthritis or rheumatism?<br>• Has a doctor ever told you that you have osteoporosis?	0. No<br>1. Yes	
	10. Chronic heart diseases	0. No	

	• Has any doctor ever told you that you had a heart attack?<br>• Has any doctor ever told you that you have angina pectoris?<br>• Has any doctor ever told you that you have a heart failure?	1. Yes	
	11. Chronic oral conditions by collapsing:<br>• Has your dentist ever told you that you have/had gum disease (periodontal disease)?<br>• Does your gum currently bleed?	0. No<br>1. Yes	

Supplementary Table S3: Distribution of various chronic conditions

Chronic conditions	India		Brazil	
	n (%)	95% CI	n (%)	95% CI
Hypertension	15,211 (29.55)	29.15-29.94	4541 (52.04)	50.96 – 53.07
Diabetes	6919 (13.44)	13.15-13.74	1364 (15.63)	14.87 – 16.40
Cancer	338 (0.66)	0.59-0.73	458 (5.25)	4.79 – 5.73
Chronic Lung diseases	3787 (7.36)	7.13-7.58	176 (2.02)	1.73 – 2.33
Chronic Heart diseases	2174 (4.22)	4.05-4.40	1005 (11.51)	10.85 – 12.20
Stroke	1063 (2.07)	1.94 – 2.19	435 (4.99)	4.53 – 5.46
Bone/joint diseases	9033 (17.55)	17.22-17.88	2494 (28.57)	27.62 – 29.53
Neurological and Psychological conditions	1108 (2.15)	2.03-2.28	1654 (18.95)	18.13-19.78
Hypercholesterolemia	1244 (2.42)	2.28-2.55	2651 (30.37)	29.40-31.34
Chronic Renal Failure	354 (0.69)	0.62-0.76	382 (4.38)	3.96 - 4.83

Oral Morbidity	7712 (14.98)	14.67-15.29	1413 (16.19)	15.42-16.97
